# Modulation of RNA splicing associated with Wnt signaling pathway using FD-895 and pladienolide B

**DOI:** 10.18632/aging.203924

**Published:** 2022-03-01

**Authors:** Deepak Kumar, Manoj K. Kashyap, Zhe Yu, Ide Spaanderman, Reymundo Villa, Thomas J. Kipps, James J. La Clair, Michael D. Burkart, Januario E. Castro

**Affiliations:** 1 Moores Cancer Center, University of California San Diego, La Jolla, CA 92093, USA; 2 ThermoFisher Scientific, Carlsbad, CA 92008, USA; 3 Amity Stem Cell Institute, Amity Medical School, Amity University Haryana, Panchgaon (Manesar), Haryana 122413, India; 4 Department of Chemistry and Biochemistry, University of California San Diego, La Jolla, CA 92093, USA; 5 CLL Research Consortium and Department of Medicine, University of California San Diego, La Jolla, CA 92093, USA; 6 Hematology-Oncology Division, Mayo Clinic, Phoenix, AZ 85054, USA

**Keywords:** splice modulation, pladienolide B, FD-895, splicing, spliceosome, intron retention, Wnt signaling

## Abstract

Alterations in RNA splicing are associated with different malignancies, including leukemia, lymphoma, and solid tumors. The RNA splicing modulators such as FD-895 and pladienolide B have been investigated in different malignancies to target/modulate spliceosome for therapeutic purpose. Different cell lines were screened using an RNA splicing modulator to test *in vitro* cytotoxicity and the ability to modulate RNA splicing capability via induction of intron retention (using RT-PCR and qPCR). The Cignal Finder Reporter Array evaluated [pathways affected by the splice modulators in HeLa cells. Further, the candidates associated with the pathways were validated at protein level using western blot assay, and gene-gene interaction studies were carried out using GeneMANIA. We show that FD-895 and pladienolide B induces higher apoptosis levels than conventional chemotherapy in different solid tumors. In addition, both agents modulate Wnt signaling pathways and mRNA splicing. Specifically, FD-895 and pladienolide B significantly downregulates Wnt signaling pathway-associated transcripts (GSK3β and LRP5) and both transcript and proteins including LEF1, CCND1, LRP6, and pLRP6 at the transcript, total protein, and protein phosphorylation’s levels. These results indicate FD-895 and pladienolide B inhibit Wnt signaling by decreasing LRP6 phosphorylation and modulating mRNA splicing through induction of intron retention in solid tumors.

## INTRODUCTION

RNA splicing and the protein machinery that guides this process, the spliceosome, constitute a very relevant biological target for cancer therapy [1, 2]. Others and we have discovered small molecule leads that modulate the spliceosome and induce apoptosis preferentially in cancer cells [1, 3]. The spliceosome macromolecules are divided into two groups: the major spliceosome, which includes U1, U2, U4, U5, U6 snRNAs, and the minor spliceosome, which comprises U11, U12 together with U5 [4, 5]. Within the major component, the SF3B core unit is comprised of spliceosome associated proteins including SF3B1 (Splicing Factor 3b Subunit 1), U2AF1 (U2 Small Nuclear RNA Auxiliary Factor 1), and SRSF2 (Serine And Arginine Rich Splicing Factor 2) that have been implicated in large a number of malignancies [6] including chronic lymphocytic leukemia (CLL) [7], uveal melanoma [8], and myelodysplastic syndrome [9]. Recent structural studies have shown that pladienolide B-related FD-895 polyketides and their analogs bind to a specific pocket within this SF3B core comprised of SF3B1, SF3B3 (Splicing factor 3B subunit 3), and PHF5A (PHD Finger Protein 5A) [10, 11]. SF3B1 and mutations in it have been exploited extensively as a therapeutic target in FLT3/ITD positive acute myeloid leukemia (AML), endometrial cancer, and hepatocellular carcinoma [12–14].

To date, a panel of splicing modulators (SPLMs) have been screened using *in vitro* and *in vivo* models for their ability to inhibit the spliceosome, and their anti-cancer properties [6]. Those compounds include FR901464 [15, 16], spliceostatin A (a derivative of FR901464) [17], thailanstatin A [18], meayamycin [19], isoginkgetin [20], sudemycin (analogs of FR901464) [21, 22], and herboxidiene [23]. The activity of SPLMs, including spliceostatin A [24], and sudemycin analogs (sudemycin C1, sudemycin D6), includes prominent induction of intron retention (IR) and exon skipping (ES) which are types of alternative RNA splicing events observed in different cell lines [25–27]. While many of these agents showed splice modulatory activity, but their primary mechanisms of inducing tumor cell death remain unknown.

E7107 (a synthetic analog of pladienolide B) entered Phase 1 clinical trials by groups at MD Anderson in Houston [28], and the Erasmus University Medical Center in Rotterdam [29]. A total of 26 patients with solid tumors were enrolled in the US-based Phase I and treated at escalating doses beginning at 0.6 mg/m^2^. E7107 was received as a 30-minute intravenous infusion on days 1 and 8 and repeated this cycle every 21 days. The selection of subsequent dose levels was performed according to accelerated design. The maximum tolerable dose (MTD) for E7107 observed was 4.3-mg/m^2^. A total of 31% of patients showed stable disease. The adverse events associated with E7107 were diarrhea, vomiting, and nausea. Blindness was observed in two patients at the 2nd and 7th cycle after receiving 3.2 mg/m^2^ and 4.3 mg/m^2^, respectively. The loss of vision event in patients led to the discontinuation of this study [28].

In the Dutch Phase, I trial, 40 patients with solid tumors were enrolled, and doses from 0.6 to 4.5 mg/m^2^ were explored. The MTD was 4.0 mg/m^2^. At 4.5 mg/m^2^, two patients experienced diarrhea of grade 4. At 4.0-4.5 mg/m^2^, dose-limiting toxicity (DLT) grade 3 diarrhea, nausea, vomiting, and abdominal cramps were observed. After drug discontinuation at 4.0 mg/m^2^, one patient experienced reversible grade 4-blurred vision. The pharmacokinetic analysis revealed a plasma half-life between 5.3 to 15.1 h. There were no complete or a partial response was observed in this trial. Severe issues with vision were observed in both trials. An increase in the pre-mRNA (intron retained) was observed in this study at MTD in the peripheral blood mononuclear cells [29]. Both trials were ultimately suspended.

In our previous studies, we explored the activity of SPLMs on normal peripheral blood mononuclear cells (PBMCs) including T and B-lymphocytes and found that SPLMs spare these cells as compared with leukemic B cells. We also reported that FD-895 and pladienolide B exhibited modulation of mRNA spicing and induced apoptosis in patient-derived CLL-B cells [30]. Here we found that FD-895 and related synthetic analogs block the G2/M phase of the cell cycle, downregulating the cyclin D1 (*CCND1*), phospho-CDC2, CDC2, and modulating PLK-1 splicing [31].

The activity of FD-895 and pladienolide B has not been evaluated in solid tumor cells lines to assess the effect of induction of intron retention on Wnt signaling pathway, therefore we designed studies to evaluate their apoptotic activity against solid tumors and the mechanisms involved in this process.

## MATERIALS AND METHODS

### Ethical statement

Informed consent was taken of the healthy donor prior to collection of blood samples from San Diego Blood Bank according to the regulation of the Institutional Review Board and Ethics committee at UC San Diego and maintained strict compliance with the Helsinki Declaration.

### Compounds

**Figure 1:**
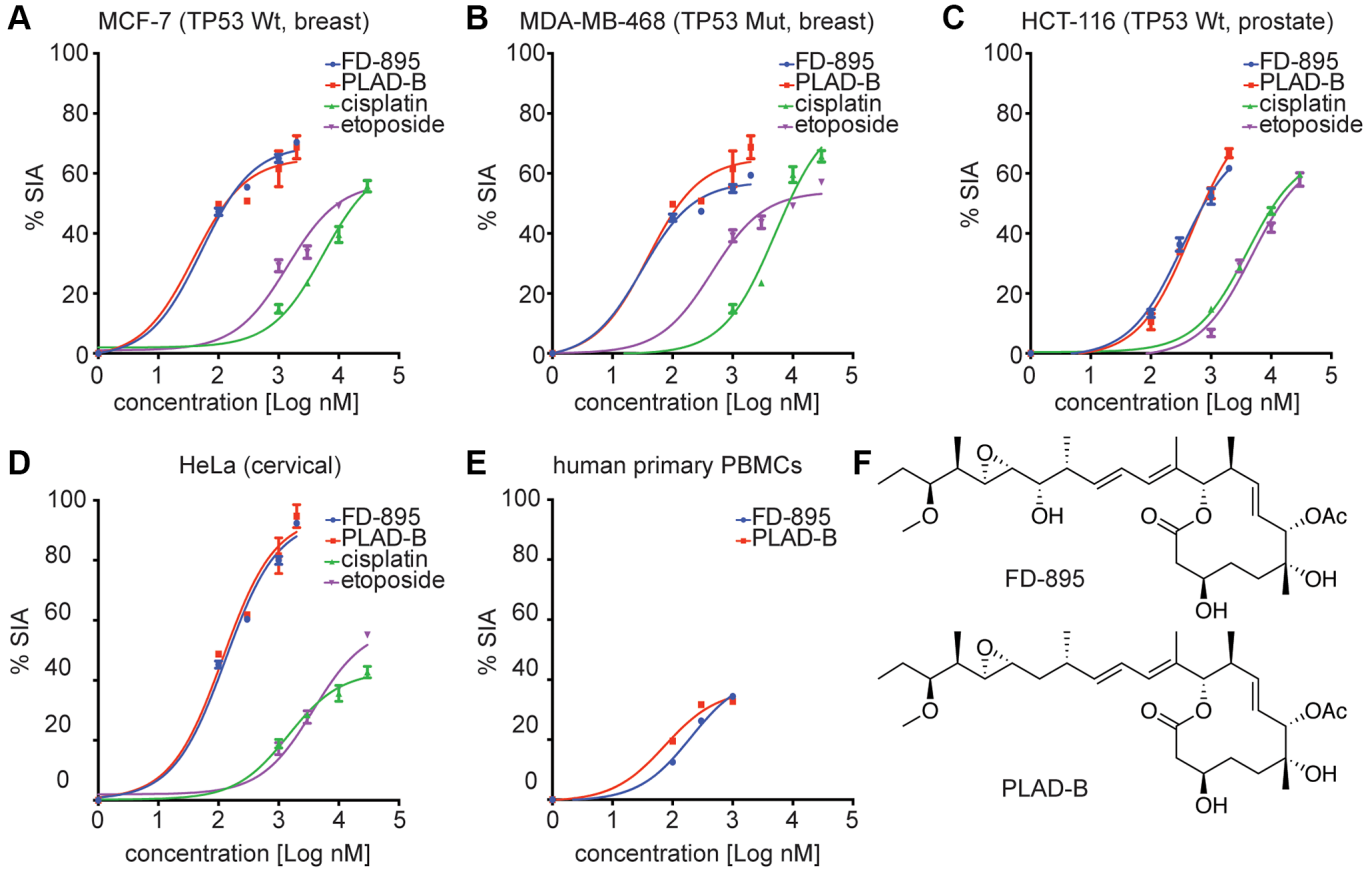
***In vitro* cytotoxicity induced by FD-895 and pladienolide B in different cancer cell lines, and normal human primary PBMCs.** Cancer Cells were exposed to FD-895 (100 nM to 2 uM), pladienolide B (100 nM to 2 uM), etoposide (1 μM to 30 μM), and cisplatin (1 μM to 30 μM) for 48 h. Apoptosis were measured in MCF-7 (**A**), MDA-MB-468 (**B**), HCT-116 (**C**) and HeLa (**D**) cells using MTS assay. The absorbance of the control (cells without treatment) was subtracted from the treated cells of each cell line. (**E**) Normal PBMC cells were exposed to FD-895, and pladienolide B. Cells were stained with propidium iodide and DiOC_6_ to differentiate dead and viable cells by using flow cytometer. Data presented in form of % specific induced apoptosis (% SIA). To assess the compound specific induced apoptosis vs. background spontaneous cell death from *in vitro* culture conditions, we calculated the percentage of SIA using the following formula: % SIA = [(compound induced apoptosis – media only spontaneous apoptosis)/(100- media only spontaneous apoptosis)] × 100. The data shows the results of samples analyzed in duplicate with the mean and its respective SD. (**F**) Structures of pladienolide-B and FD-895.

FD-895 was prepared through total synthesis [32]. Pladienolide B (sc-391691, Santa Cruz Biotechnology, Santa Cruz, CA, USA), etoposide (E1383, Sigma-Aldrich, St Louis, MO, USA) and cisplatin (479306, Sigma-Aldrich, St Louis, MO, USA) were obtained commercially (Figure 1). Oligonucleotides were purchased *via* custom synthesis (Integrated DNA Technologies).

### Cell culture methods

The MCF-7 (RRID:CVCL_0031), MDA-MB-468 (RRID:CVCL_0419), HS578T (breast cancer, RRID:CVCL_0332), A2780 (RRID:CVCL_0134), SKOV3 (ovarian cancer, RRID:CVCL_0532), 786-O (renal adenocarcinoma, RRID:CVCL_1051), HeLa (Cervical cancer, RRID:CVCL_0030), and HEK-293 cell lines were obtained from ATCC. MCF7 was cultured in DMEM (catalog # 12800017, GIBCO, Grand Island, USA) + 10% fetal bovine serum (FBS, catalog # FB-02, Omega Scientific, Tarzana, CA, USA) +2 mM L-glutamine and 1% Pen/Strep (catalog # 15140148, Invitrogen Corporation, CA, USA) supplemented with 0.01 mg/mL human recombinant insulin. Other cell lines were maintained in DMEM supplemented with 10% FBS, 2 mM L-glutamine, and 1% of Pen/Strep. Additionally, two ovarian cancer cell lines with differential cisplatin sensitivity, OV-2008 (sensitive, RRID:CVCL_0473) and it’s resistant variant C13 were obtained from Prof. Stephen Howell (UC San Diego). To complete the set, a final colon cancer cell line HCT116 (RRID:CVCL_0291) was obtained from the Johns Hopkins School of Medicine, Baltimore, MD. The suspension cell lines including Jeko-1, JVM2, and Mino cell lines were cultured in RPMI-1640 (catalog # R7509, Sigma) supplemented with 10% FBS along with 1% Pen-Strep. All cell lines were incubated at 37°C in an atmosphere of 5% CO_2_ and routinely monitored for Mycoplasma infections by PCR analyses.

### Flow cytometry analyses

Normal PBMCs were treated with FD-895 (100 nM to 2.0 μM), and pladienolide B (100 nM to 2.0 μM), for 48 h. Cell viability was determined by flow cytometry after staining with conventional live staining with 40 μM 3,3′dihexyloxacarbocyanine iodide (DiOC_6_; Life Technologies, Carlsbad, CA, USA) and 15 μM (10 μg/mL) of propidium iodide (PI; Sigma-Aldrich, St Louis, MO, USA). Data were analyzed by using FlowJo software (version 6.4.7; Tree Star). Using this assay, viable cells excluded PI and stained brightly positive for DiOC_6_ as it targets metabolically active mitochondria of alive cells [30–33].

### Calculation of % specific induced apoptosis (SIA)

To discriminate the compound specific induced apoptosis vs. background spontaneous cell death from *in vitro* culture conditions, we calculated the percentage of specific induced apoptosis (% SIA) using the following formula: >% SIA = [(compound induced apoptosis – media only spontaneous apoptosis)/(100- media only spontaneous apoptosis)] × 100.

### Cell proliferation assays

The cell proliferation assays were conducted in adherent cell lines (HCT116, MCF-7, MDA-MB-468, HS578T, OV-2008, A2780, SKOV3, 786-O, HEK-293, and HeLa) by using CellTiter 96 AQueous non-radioactive colorimetric method (G5421, Promega, Madison, WI, USA). Briefly, a total of 3000 cells/well were seeded in a 96-well flat-well plate followed by treatment with FD-895 (100 nM to 2.0 μM), pladienolide B (100 nM to 2.0 μM), cisplatin (1 μM to 30 μM) or etoposide (1 μM to 30 μM) in triplicate for 48 h at 37°C. Following the incubation, 20 μL of CellTiter 96 AQueous solution (Promega, Madison, WI, USA) was added directly to each well. Non-treated cells were considered as the control. After staining, the plates were incubated for an additional 2 h and then read on a 96-well plate reader (Molecular Devices, Sunnyvale, CA, USA). Absorbance readings were recorded absorbance at 490 nm using empty wells (air) for background collection.

### Reverse transcriptase PCR (RT-PCR) analyses

HCT116, MCF-7, MDA-MB-468, HeLa, Jeko-1, JVM-2 and Mino cells (10^6^ cells/well) were treated with 100 nM FD-895, 100 nM pladienolide B, 30 μM cisplatin or 30 μM etoposide for 4 h. RNA isolation was done using mirVana miRNA Isolation Kit (Ambion, Austin, Texas). The 200 ng of RNA was subjected to DNase I (Life Technologies, Carlsbad, CA, USA). The cDNA was prepared by using SuperScript III Reverse Transcriptase Kit (Life Technologies, Carlsbad, CA, USA), and PCR reactions were performed in 20 μL of reaction volume. PCR conditions were 95°C for 3 min; 35 cycles of 95°C for 30 s, 58°C for 30 s, and 72°C for 45 s; followed by 72°C for 5 min. PCR products were separated on a 2% agarose gel and stained with ethidium bromide. Details of the primers used for RT-PCR are described in Table 1.

**Table 1:** Sequences of primers used in the RT-PCR.

**Primer**	**Sequence**
*DNAJB1*-FW	5′-GAACCAAAATCACTTTCCCCAAGGAAGG-3′
*DNAJB1*-RV	5′-AATGAGGTCCCCACGTTTCTCGGGTGT-3′
*RNU6A*-FW	5′-CGCTTCGGCAGCACATATAC-3′
*RNU6A*-RV	5′-GAATTTGCGTGTCATCCTT-3′
*LEF1*-*FP*	5′-AGGAACATCCCCACACTGAC-3′;
*LEF1*-*RP*	5′-AGGTCTTTTTGGCTCCTGCT-3′
*CCND1*-*FP*	5′-AATGACCCCGCACGATTTC-3′
*CCND1*-*RP*	5′-TCAGGTTCAGGCCTTGCAC-3′
*FN1*-FP	5′-ACCTACGGATGACTCGTGCTTT-3′
*FN1*-RP	5′-TTCAGACATTCGTTCCCACTCA-3′
*GSK3*β-FP	5′-ATCAAGGCACATCCTTGGAC-3′
*GSK3*β-RP	5′-CAATTGCCTCTGGTGGAGTT-3′
*LRP5*-FP	5′-GCCTGCAACAAGTGGACA-3′
*LRP5*-RP	5′-CCTGCAGCACTATGTCTGTGA-3′

FP and RP denote forward and reverse primers respectively.

### Quantitative reverse transcriptase-PCR (qRT-PCR) analyses

The HeLa cells were treated with 100 nM FD-895 or 100 nM pladienolide B for 6 h, 12 h, or 24 h, and the RNA isolation and cDNA preparation were done as described above. The amounts of mRNA of *LEF1* (Lymphoid enhancer-binding factor-1), *FN1* (fibronectin 1), and *CCND1* genes were determined using Power SYBR Green PCR master mix (Applied Biosystems, Foster City, CA) real-time qRT-PCR using specific primers [34]. PCR was conducted using 5 picomoles of each primer and 20 ng of the obtained cDNA. PCR conditions were 50°C for 2 min; 95°C for 10 min; 40 cycles of 94°C for 15 s, and 60°C for 1 min. The mRNA levels were calculated using the 2^−ΔΔCT^ method [35]. *GAPDH* was used as a control for normalization.

### Pathway reporter arrays

Cignal Finder Reporter Array (336821, Qiagen/SABiosciences, Frederick, MD, USA) was used to assess 45 different signaling pathways. HeLa cells were seeded into wells (50,000 cells/well) of the Cignal Finder 96-well plates (CCA-901L, Qiagen, SABiosciences, Frederick, MD, USA) for introducing pathway reporters into cells by reverse transfection according to the manufacturer’s protocol. Briefly, reporter DNA constructs in each plate well were re-suspended with 50 μL Opti-MEM and then mixed with 50 μL diluted Lipofectamine 2000 transfection (Life Technologies, Carlsbad, CA, USA) reagent. Cells were suspended in Opti-MEM (Life Technologies, Carlsbad, CA, USA) supplemented with 10% of FBS and 0.1 mM non-essential amino acids at a density of 6 × 10^5^ cells/mL, and then 50 μL of the cell suspension was added into each plate well and mixed with DNA resident in the plate and added transfection reagent. The cells were incubated for 3 h. Following transfection; the cells were treated with vehicle (Opti-MEM) or 100 nM FD-895 for 3 h in Opti-MEM media. Luciferase and renilla expression were determined (Qiagen/SABiosciences Corp., Frederick, MD, USA).

### Western blot analyses

HeLa cells were treated with 100 nM FD-895 or 100 nM pladienolide B for 12 h, 24 h, and 48 h for β-catenin, LEF1, LRP6 (LDL Receptor Related Protein 6), and phospho-LRP6. The cells were then washed with PBS (2 × 5 mL) and lysed with modified RIPA buffer at 4°C. Untreated cells were used as a control. The whole-cell protein was quantified according to the Bradford method [36]. Lysates in sample buffer (2% Sodium dodecyl sulfate (SDS), 10% glycerol, 80 mM Tris•HCl (pH 6.8), 720 mM β-mercaptoethanol and 0.001% bromophenol blue) were denatured at 95°C for 5 min. Total cellular proteins (30 μg *via* Bradford analyses) were subjected to SDS-polyacrylamide gel electrophoresis (PAGE) using a 4–20% Criterion Precast Gel (Bio-Rad, Hercules, CA), and the proteins were transferred to polyvinylidene difluoride (PVDF) membrane (Millipore, Bedford, MA). After blocking with 5% bovine serum albumin (BSA) for 1 h in Tris-buffered saline, 0.1% Tween 20 (TBST, 20 mM Tris•HCl, 137 mM NaCl, 0.1% Tween-20 pH 7.6), the membrane was incubated with the following primary antibody overnight at 4°C. The primary antibodies include rabbit mAb anti-LEF1, rabbit anti-Phospho-LRP6 (Ser1490), rabbit anti-LRP6, rabbit mAb anti-β-catenin, and mouse Ab anti-β-actin were obtained from Cell Signaling Technology (Beverly, MA) and used at a dilution of 1:1000. After primary mAb staining and washing thrice with TBST, the membranes were incubated with HRP-labeled anti-rabbit (sc-2030, Santa Cruz Biotechnology) or HRP-labeled anti-mouse (sc-2031, Santa Cruz Biotechnology) secondary antibodies with a dilution of 1:5000 dilutions for 40 min at rt. After incubation, the membrane was washed thrice with TBST and developed using an enhanced chemiluminescence (ECL) kit (Pierce Thermo Scientific Inc., Rockford, IL).

### Bioinformatics analysis

Gene-gene interaction networks were predicted and generated with GeneMANIA (Gene Multiple Association Network Integration Algorithm) available at http://genemania.org [37].

### Statistical analysis

The data presented as mean ± standard deviation (SD). The data was analyzed using GraphPad Prism 6.0 (GraphPad Software, La Jolla, CA). Multiple groups were compared using Bonferroni correction and *p* < 0.05 was considered statistically significant.

### Ethics approval and consent to participate

The study involved human, animal or cell lines as a material for experimental purpose and the ethical clearance was conducted before starting the study.

### Consent for publication

All authors consent to the publication of the manuscript in "Aging". Further, figures or tables are original, so there was no requirement of taking permission or consent from anyone.

### Availability of data and material

All data generated and analyzed during our study are included in the published article.

## RESULTS

### *In vitro* cytotoxicity evaluation of FD-895 and pladienolide B in colon, breast, cervical cancer cell lines

We previously reported the apoptotic activity of FD-895 and pladienolide B in CLL-B, mantle cell lymphoma (MCL), and other B and T lymphoma cell lines [31] Further, we were interested in exploring the apoptotic activity of FD-895 and pladienolide B in different solid tumor cell lines (Figure 1A–1D). The chemical structure of both SPLMs has been shown in Figure 1F. Using an expanded panel of cell lines, we found that FD-895 and pladienolide B IC_50_ ranged from 30.7 ± 2.2 to 415.0 ± 5.3 nM (Table 2) across the breast, colon, and cervix tumor cell lines (Figure 1A–1D). Upon treatment of normal PBMCs with FD-895 and pladienolide B, the lack of activity (IC_50_ values <450 nM) was not achieved in normal PBMCs, an observation that suggests that both the FD-895 and pladienolide B spare the normal PBMCs, but not the leukemic B cells (Figure 1E) [30]. We also tested the normal cell line HEK-293 in response to FD-895 (100 nM to 2 μM) and found that there was non-significant cell death induced with varying concentrations of FD-895 when compared with control (*p* > 0.05), but cisplatin (30 μM) induced significant cell death in HEK-293 cells (*p* < 0.05, Supplementary Figure 1).

**Table 2:** IC_50_ values for FD-895 and pladienolide B in selected tumor cell lines.

**Cell lines**	**Type of Cancer**	**FD-895 (nM)**	**Pladienolide B (nM)**
HCT116 (*TP53*, +/+)	Colon	34.1 ± 2.4	42.3 ± 3.1
HCT116 (*TP53*, +/−)	Colon	75.2 ± 2.5	65.6 ± 2.9
HCT116 (*TP53*, −/−)	Colon	100.7 ± 1.2	88.6 ± 2.0
MCF-7 (*TP53*, +/+)	Breast	51.7 ± 1.9	38.7 ± 3.8
MDA-MB-468 (*TP53*, +/−)	Breast	30.7 ± 2.1	38.5 ± 3.8
HS578T (*TP53*, −/−)	Breast	139.7 ± 1.1	112.0 ± 3.1
OV-2008	Ovarian	311.6 ± 2.2	344.5 ± 1.3
A2780	Ovarian	415.0 ± 5.2	337.0 ± 7.0
SKOV3	Ovarian	143.4 ± 2.1	128.7 ± 3.2
786-O	Renal	412.5 ± 2.4	293.6 ± 3.1
HeLa	Cervical	131.0 ± 3.3	118.4 ± 4.4

### Cytotoxic evaluation of FD-895 and pladienolide B in ovarian cancer cells regardless of differential cisplatin sensitivity

**Figure 2:**
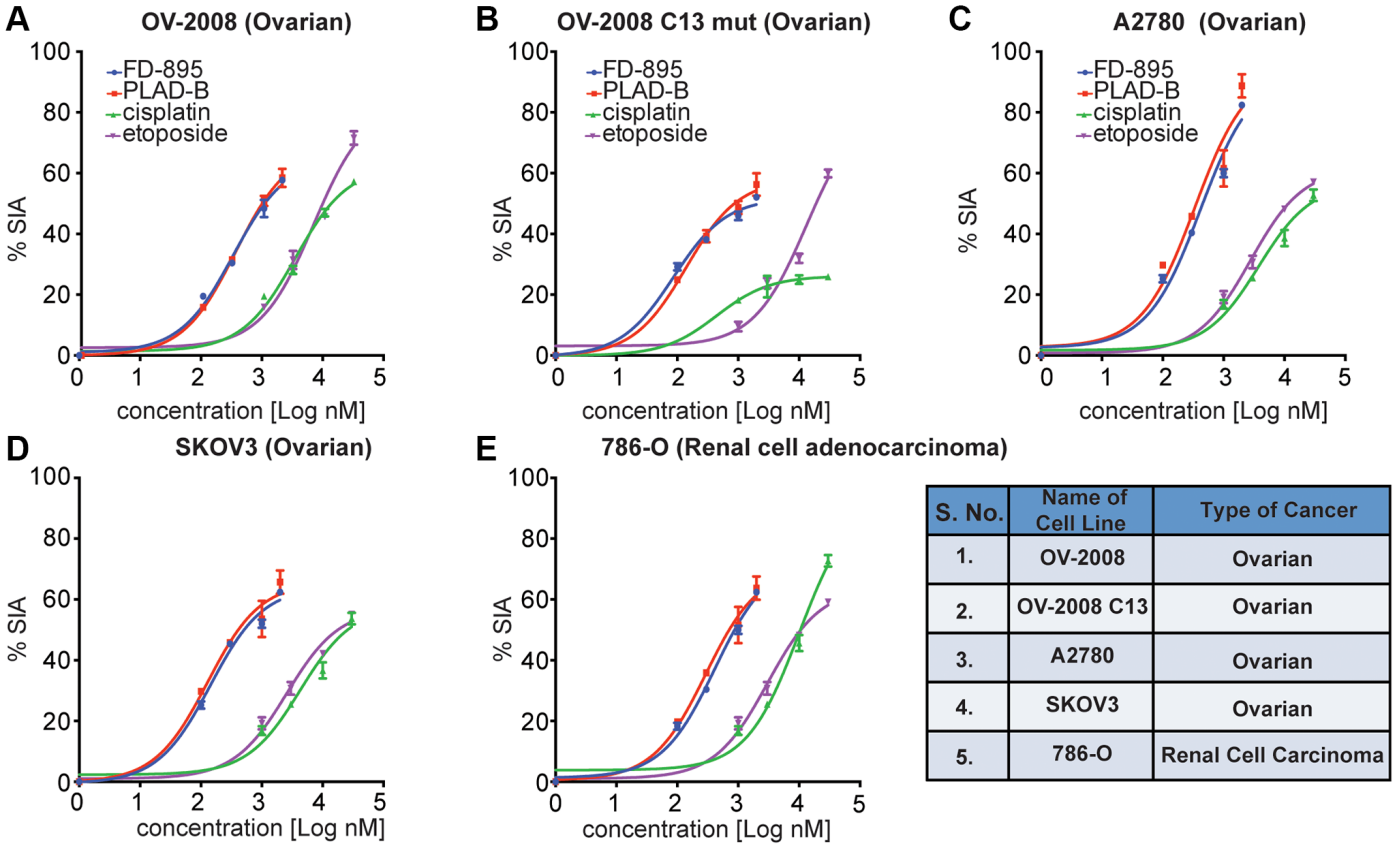
**FD-895 or by pladienolide B induced apoptosis in ovarian and renal cancer cells.** Ovarian cancer cells (**A**) OV-2008, cisplatin sensitive (**B**) OV-2008 C13 mut, cisplatin resistant, (**C**) A2780, (**D**) SKOV3 or (**E**) renal cancer cells (786-O) were incubated with FD-895 (100 nM to 2 μM), pladienolide B (100 nM to 2 μM), etoposide (1 μM to 30 μM), or of cisplatin (1 μM to 30 μM) for 48 h. Cells viability were measured as using MTS assay. This experiment was repeated in triplicate independently. Data presented in form of % SIA. The data shows the results of samples analyzed in duplicate with the mean and its respective SD.

Next, we turned our attention to explore the activity of splice modulation on cell lines displaying sensitivity or resistance to cisplatin, as the latter is an issue in the treatment of solid tumors including ovarian [38], cervical cancer [39], gastric adenocarcinoma [40], prostate cancer [41], colorectal [42], and head and neck squamous cell carcinoma [43]. Here, we used two human ovarian cancer cell lines, one consisting of a cisplatin-sensitive parental line, OV2008, and the other stably cisplatin-resistant subline, OV2008/C13 derived by *in vitro* selection with cisplatin. We began by screening these cell lines for their induction of apoptosis when treated with FD-895, pladienolide B, cisplatin, or etoposide. FD-895 and pladienolide B induced significant apoptosis in both parental and cisplatin-resistant OV2008 cells (Figure 2A, 2B). We also observed that FD-895 and pladienolide B demonstrated significant apoptosis in A2780 and SKOV3 ovarian cancer cell lines as compared to cisplatin and etoposide (Figure 2C, 2D). These findings suggest that nanomolar concentrations of SPLMs have the potential to overcome cisplatin resistance. We also evaluated the apoptotic activity of FD-895 and pladienolide B in 786-O (renal) cancer cells. We found that both splice modulators were ten-fold more efficient at induced apoptosis than cisplatin and etoposide (Figure 2E).

### FD-895 and pladienolide B induced spliceosome modulation marked by intron retention in cancer cells

**Figure 3:**
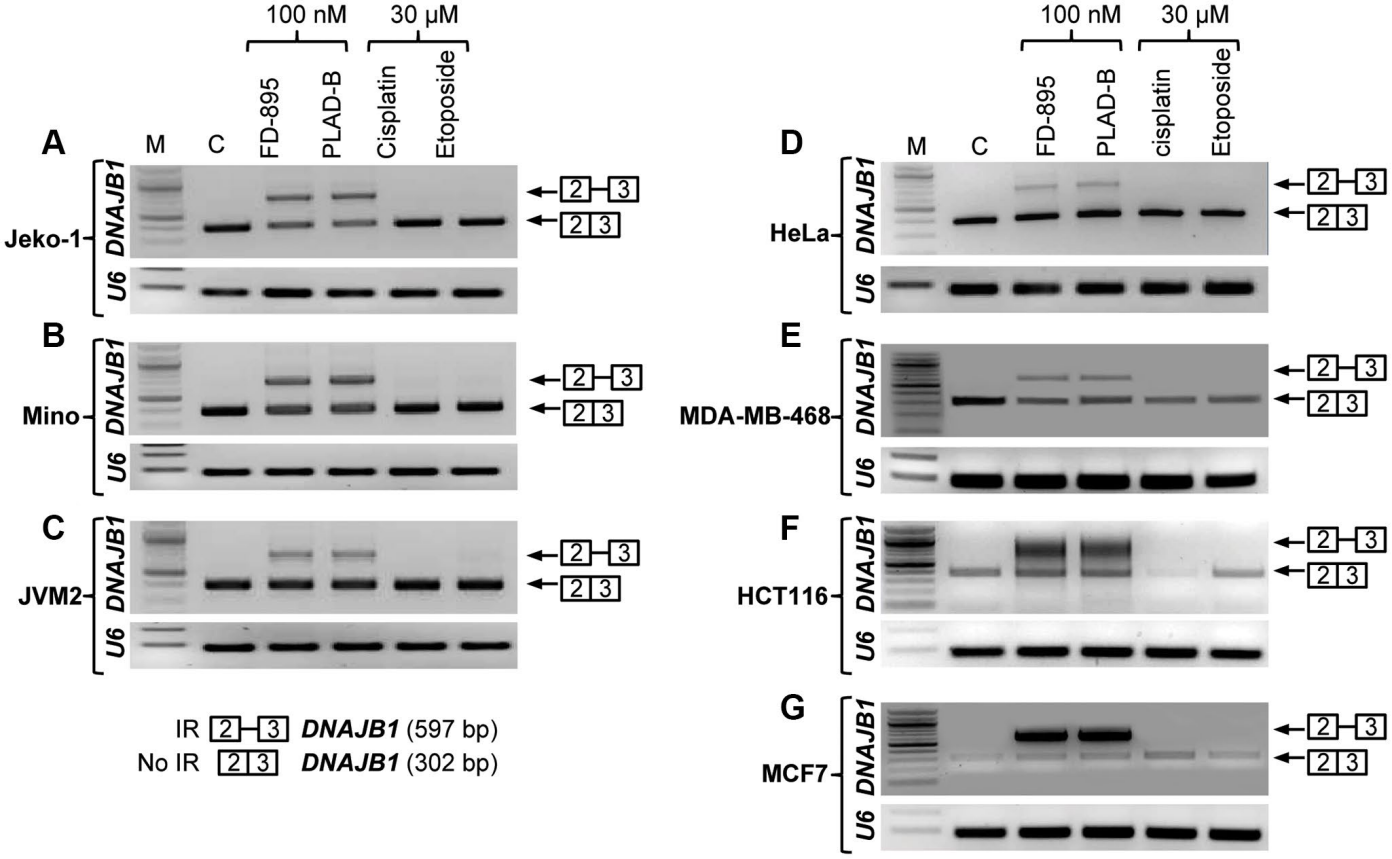
**Intron retention of *DNAJB1* gene in different cancer cell lines.** Tumor cell lines were treated with 100 nM of FD-895, 100 nM pladienolide B, 30 μM cisplatin or 30 μM etoposide for 4 h. Analysis of IR of *DNAJB1* mRNAs was evaluated by RT-PCR in (**A**–**C**) Mantle cell lymphoma cells, (Jeko-1, Mino and JMV-2), (**D**) HeLa, (**E**) MDA-MB-468 (**F**) HCT116 and (**G**) MCF-7 cells. *RNU6A*, an intronless gene was used as RNA quality and loading control.

In previous studies, we found that FD-895 and pladienolide B induced IR in CLL and MCL cells [30–32]. Here, we expand our understanding of their ability to induce IR across an expanded panel of cancer cell lines, including Jeko-1, Mino, JVM2, HeLa, HCT116, MCF-7, and MDA-MB-468 cell lines. In brief, we incubated 10^6^ cells/well from each cell line with a 100 nM FD-895, 100 nM pladienolide B, 30 μM cisplatin or 30 μM etoposide for 4 h. After treatment, the levels of spliced and unspliced gene expression were evaluated by RT-PCR. We observed that cells treated with FD-895 or pladienolide B demonstrated IR, which was not observed with non-splice modulatory controls, cisplatin, or etoposide (Figure 3) using *DNAJB1* (DnaJ Heat Shock Protein Family (Hsp40) Member B1) as a surrogate marker for spliceosome modulation [30]. IR was observed in cells treated with FD-895 or pladienolide B for *DNAJB1* when compared to the intronless gene *RNU6A* used as a loading control RNA (Figure 3A–3G).

### FD-895 downregulates and modulates splicing of proteins involved in Wnt signaling

**Figure 4:**
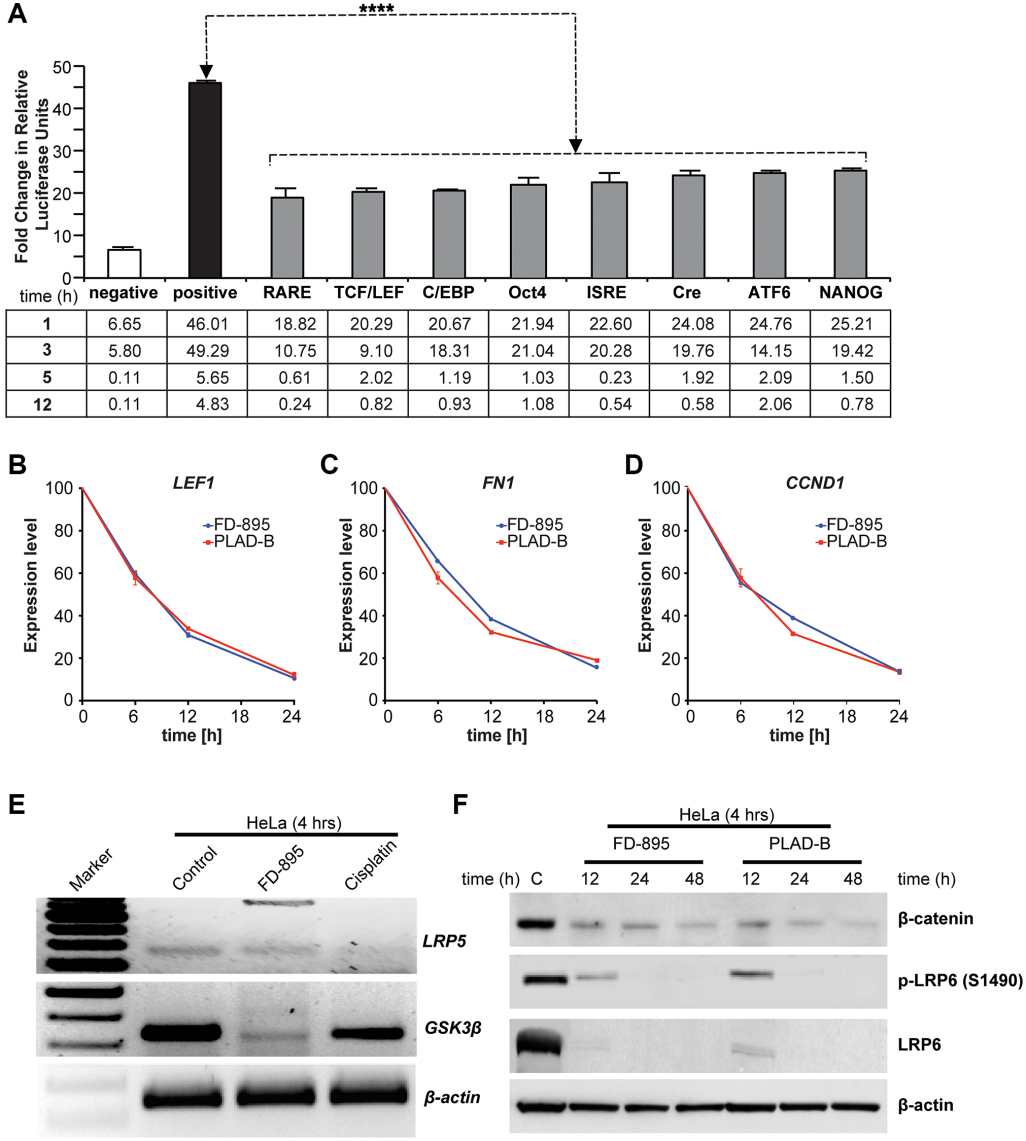
**Effect of FD-895 on different pathways and effect on Wnt signaling in HeLa cells.** (**A**) HeLa cells were use for introducing pathway reporters into cells *via* reverse transfection. Post-transfection, the cells were treated with vehicle or 100 nM FD-895 for 3 h. Luciferase and renilla expression was evaluated. HeLa cells were exposed to 100 nM of FD-895 or 100 nM pladienolide B for 6 h, 12 h or 24 h and expression of (**B**) *LEF1*, (**C**) *FN1*, and (**D**) *CCND1* were determined by qRT-PCR. (**E**) HeLa cells were treated with 100 nM of FD-895 or 30 μM cisplatin for 4 h. Analysis of IR for *GSK3β* and *LRP5* mRNAs was evaluated by using RT-PCR. (**F**) HeLa cells were treated with 100 nM of FD-895 or 100 nM pladienolide B for 6 h, 12 h or 24 h. Protein extracts were immunoblotted for β-catenin, phohspho-LRP6, LRP6, and β-actin.

To investigate the intracellular signaling pathways affected by FD-895, we applied the Cignal 45-Pathway Reporter Array to simultaneously analyze FD-895 effect on 45 different signaling pathways [44]. HeLa cells, selected due to their SPLM sensitivity, were treated with FD-895 over 12 h and baseline-signaling profile was compared to vehicle control (Figure 4A). Interestingly, treatment with 100 nM FD-895 modulated a number of pathways including notch signaling, octamer-binding transcription factor 4 (OCT4), activating transcription factor 6 (ATF6), NANOG, and Wnt signaling pathway as early as within 1 h but decreased as the time progressed (Figure 4A).

Among these pathways, we found that the Wnt signaling pathway was activated at an early time point but downregulated as the duration of FD-895 prolonged, as monitored by the TCF/LEF reporter in cervical cancer cell line HeLa (Figure 4A).

Alternative RNA splicing events have been reported within the Wnt signaling pathway for various molecules such as in LRP6, ROR, Axin, APC, CK1, and Catenin, beta-1 (CTNNB1) [45]. We selected Wnt signaling pathway not only because of this but also because Wnt5A overexpression has been reported in multiple cancers and it is associated with disease progression, metastasis, and resistance to treatment [46]. Additionally, there were several reasons to select LRP5 and GSK-3β for further validation. We know that both lipoprotein receptor-related protein (LRP) 5 and 6 are crucial Wnt co-receptors and interact with other components of the Wnt signaling pathway. Additionally, both LRP5 and LRP6 are oncogenic proteins as well. Similarly, GSK-3β, a serine-threonine kinase and a negative regulator of the oncogenic Wnt/β-catenin signaling pathway [47]. Moreover, the role of Wnt signaling has been well established in CLL [34], and in our previous study, in the RNAseq data analysis, we found that both LRP5 and GSK-3β were found to have 3.44, and 5.1-fold IR, respectively in CLL-B cells treated with splice modulator compared with control CLL [30].

From this data, we selected Wnt signaling pathway to study further because as Wnt5A overexpressed and has been correlated with cervical carcinoma [46]. We began by exploring its effect on the TCF/LEF reporter system. As shown in Figure 4A, we observed that TCF/LEF reporter showed downregulation in 100 nM FD-895 treated HeLa cells after 12 h. We also investigated the effect of FD-895 and pladienolide B on mRNA expression of selected genes *LEF1*, *FN1*, and *CCND1* [48–50] which involved in the Wnt/β-catenin signaling pathway. The HeLa cells were exposed to 100 nM of FD-895 or pladienolide B for different times and *LEF1*, *CCND1*, and *FN1* mRNA levels measured by qRT-PCR. The expression of *LEF1*, *CCND1*, and *FN1* was significantly decreased by 8.33, 6.25, and 5.26 fold respectively (Figure 4B–4D) as the incubation period increased with the maximum level appearing at 20% at 24 h. Further, we performed the RT-PCR to detect splicing in genes involved in Wnt signaling pathway like *GSK3β* and *LRP5*. HeLa cells treated with 100 nM FD-895 showed weak IR in *GSK3β* and *LRP5*, an observation that was not observed in non-splicing controls (30 μM cisplatin). In comparisons to chemotherapy, the macrolides significantly downregulated the expression of *GSK3β* in HeLa cell line (Figure 4E). We then conducted Western blot analyses to study the correlation between mRNA and protein. Treatment with 100 nM FD-895 or 100 nM pladienolide B treatment resulted in the downregulation of β-catenin, LEF1, total LRP6, and phospho-LRP6 protein levels (Figure 4F). Altogether, the results from qRT-PCR and Western blot data demonstrate downregulation of key Wnt signaling pathway molecules in the HeLa cell line.

### Bioinformatics based gene-gene interaction

**Figure 5:**
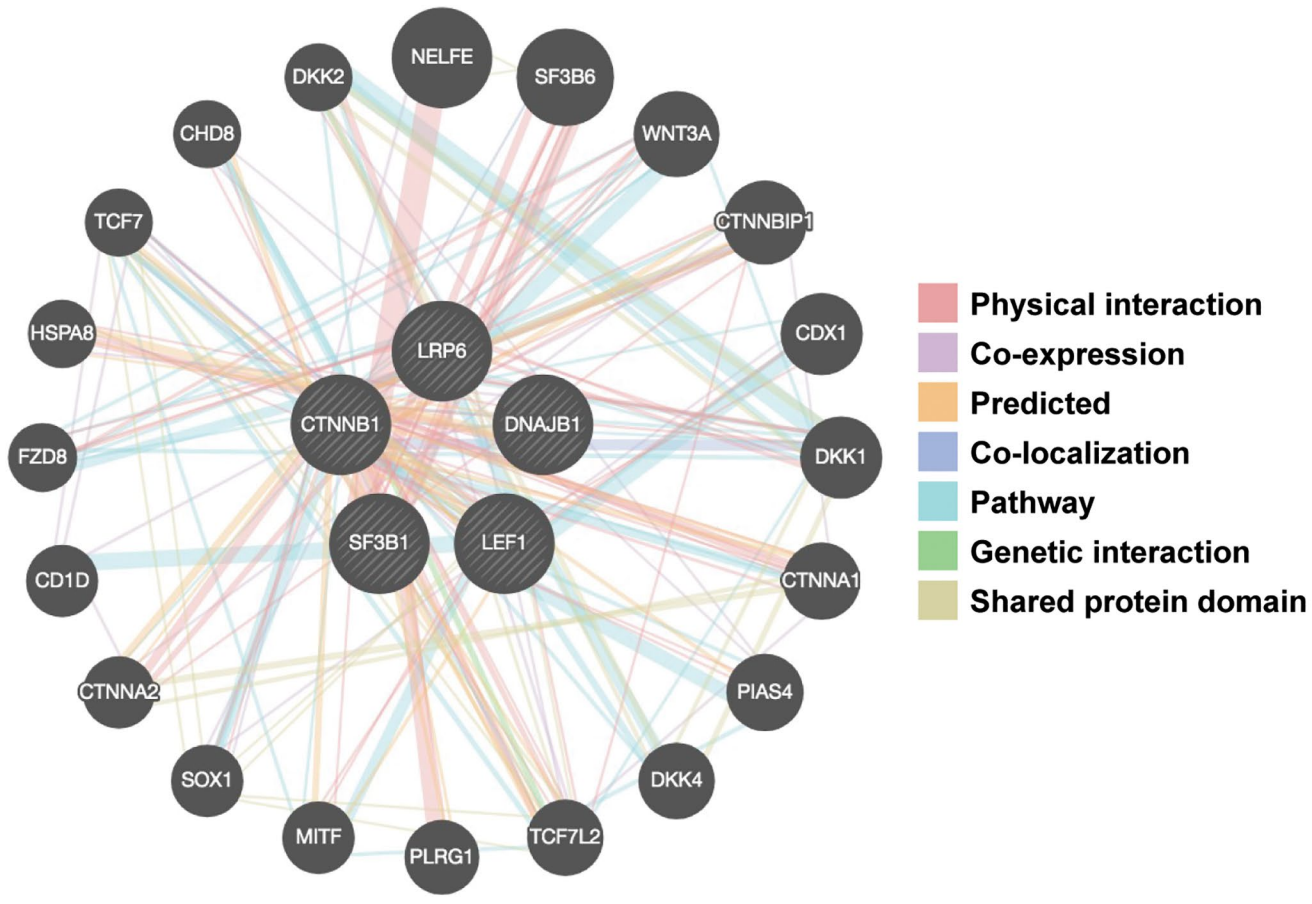
**Gene-gene interaction networks among selected genes constructed by GeneMANIA.** A gene-gene network was constructed with the search tool for the retrieval of interacting genes available in GeneMANIA annotation information for selected 5 genes, including physical interaction, genetic interaction, co-expression and shared pathways and protein structure domain. The central black nodes denote 5 selected genes used as an “INPUT”, and peripheral nodes denote gene interactions with the black nodes. The network also contains 20 normal human genes. The size of the circles indicates the degree of interaction.

Our next studies explored the effects of FD-895 and pladienolide B on select gene-gene interaction networks using GeneMANIA. We used *DNAJB1*, *LEF1*, *CTNNB1*, *LRP6*, and *SF3B1* as “Input” genes for gene-gene interaction analysis and included *SF3B1* in this analysis because SF3B1 is a component of the spliceosome-binding pocket of pladienolide B [51, 52]. As shown in Figure 5, we found the selected genes were part of the network directly or indirectly associated with *SF3B1*.

We obtained total twenty genes from GeneMANIA analysis and among those top ten interactor molecule emerge in the network were based on the size of the circle including *NELFE* (negative elongation factor complex member E, rank-1), SF3B6 (splicing factor 3b subunit 6, rank-2), *Wnt3A* (Wnt family member 3A, rank-3), *CTNNBIP1* (catenin beta interacting protein 1, rank-4), *CDX1* (caudal type homeobox 1, rank-5), *DKK1* (dickkopf Wnt signaling pathway inhibitor 1, rank-6), *CTNNA1* (catenin alpha 1, rank-7), *PIAS4* (protein inhibitor of activated STAT 4, rank-8), *DKK4* (dickkopf Wnt signaling pathway inhibitor 4, rank-9), and *TCF7L2* (transcription factor 7 like 2, rank-10). Among these genes few genes are involved in Wnt signaling such as Wnt3A, CTNNA1, DKK4, and TCF7L2 suggest that there is an important role of RNA splicing machinery in regulation of Wnt signaling pathway.

## DISCUSSION

Splice modulation offers a unique opportunity to selectively modulate ongoing rapid cellular growth, and has shown early promise as a therapeutic target [53, 54]. Leveraging methods developed in prior studies in CLL, we tested *in vitro* activities of FD-895 and pladienolide B in solid tumor and mantle cell lymphoma cells. Here, our studies show that FD-895 and pladienolide B show potent apoptotic activity at nanomolar concentrations across the majority of cell lines screened. Both splice modulators demonstrated significant induction of apoptosis in human ovarian cancer cells, OV2008 and C13 (cisplatin resistant), and renal carcinoma.

Cell lines with mutant TP53 showed higher IC_50_ with FD-895 and pladienolide B. In the past study by our group, we observed that in the case of CLL patients harboring 17pDEL/*TP53* mutations harboring CLL patients, the splice modulators were able to induce *in vitro* cytotoxicity suggesting that the induction of cell death was TP53 independent. Splice modulators such as TG003 interfere with in the splicing machinery leading to TP53 activation, which induces TP53 accumulation, elevated p53 transcriptional activity, and p53-dependent G_1_ cell cycle arrest in U2OS (human osteosarcoma) and A375 (human melanoma) cell lines [55]. Further, in hepatocellular carcinoma (HCC), *TP53* splice mutations have been associated with the development and progression of the disease [56]. These observations may differ in different malignancies due to behavior in the pathophysiology and the driving receptor/signaling pathway. This is important to note that not only studying alternative RNA splicing events such as IR, ES and A5’SS, A3’SS is crucial but the splice mutations and their impact on the development and pathophysiology of cancer could be of utmost significance.

Interestingly, FD-895 and pladienolide B spares and normal cell inducing PBMCs (include T cells, B cells, and NK cells, monocytes, and dendritic cells), suggesting that these compounds have a preference to induce *in vitro* cytotoxicity preferentially in cancer cells and this creates a beneficial therapeutic window for patient treatment. These findings were in concordance with our previous observations in normal B cells [30]. We also tested the normal cell line HEK-293 in response to FD-895 and found no significant cell death, which was different to the cytotoxicity induced by cisplatin. These data further corroborated our observation that SPLMs induce prudentially apoptosis in tumor cell lines, but not in normal cells [57, 58].

In other studies as well, HEK-293 cell line was also tested *in vitro* in response to isoginkgetin (splicing modulator control), a bioflavonoid derived from the leaves of *Ginkgo biloba*. In those studies, the prop-apoptotic activity induced by SPLMs including the isoginkgetin was significantly lower [22, 59, 60].

Following *in vitro* cytotoxicity assays, we assessed if RNA splicing modulators induce IR in the different solid tumor as well as in mantle cell lymphoma cells. Using *DNAJB1* has been used as a surrogate marker of splicing modulation; we found that FD-895 as well as pladienolide B induced IR in *DNAJB1* in all the cell lines at 100 nM [30, 32].

Next, we turned our attention to identify the specific pathways regulated by these splice modulatory events. In HeLa cells, we observed that treatment with 100 nM of FD-895 resulted in modulation of ATF6-C/EBP-β-signaling (ATF6 and C/EBP response elements), Wnt signaling (TCF/LEF reporter), type 1 interferon-induced signal transduction (ISRE reporter), CREB signaling (cAMP response element, CRE is the response element), NANOG, and OCT4 pathways. The following discussion provides a brief overview of the significance of these pathways in their response to splice modulation by FD-895.

OCT4 is a homeodomain transcription factor of the POU family also known as POU5F1 (POU domain, class 5, transcription factor 1). It is involved in self-renewal of undifferentiated embryonic stem cells and therefore used as a marker for dedifferentiation [61]. OCT4 is crucial for determination of fates of the inner mass and embryonic stem cells. OCT4 is capable of maintaining pluripotency throughout the embryonic development. It is also involved in proliferation of cancer cells including pancreatic, liver, testicular and lung cancer of adult germ cells [62].

In ATF6 pathway, the central player is ATF6 that works in a concentration dependent manner. At low levels, ATF6 activates the unfolded protein response (UPR) for self-defense. At high levels, it mediates apoptosis. ATF6 is crucial for transition from self-defense to self-destruction of cells in endoplasmic reticulum (ER) stress [63].

In NANOG pathway, the NANOG transcription factor is important as it is involved in self-renewal regulation as well as maintenance of the embryonic stem cell pluripotency [64]. NANOG has been reported in number of malignancies including leukemia [65]. Both NANOG and OCT4 pathways’ are involved in regulation of pluripotency of stem cells [66].

We observed modulation of T-cell factor/lymphoid enhancer factor (TCF/LEF) in response to treatment with FD-895 in HeLa cell. Wnt alone led to an accumulation of β-catenin in the cytoplasm, but its nuclear activity is largely mediated by TCF/LEF only. TCF/LEF is an important component of Wnt or Wnt/β-catenin signaling pathway. The Wnt/β-catenin signaling has been reported in a number of malignancies including cervical cancer [67, 68]. Interestingly, we found that Wnt signaling pathway was downregulated after 12 h of FD-895 treatment. In human cells, Wnt is a secreted protein that acts as a ligand for ROR1 a receptor tyrosine kinase [69–71]. Wnt/β-catenin activation occurs upon binding of Wnt5A with membranous proteins Frizzled (FZ) receptor and lysophosphatidic acid receptors (LRP5/6) protein. This binding event leads to recruitment of the scaffolding protein Disheveled (DVL), which results in phosphorylation of LRP5/6 receptors. During the course of these studies, we found that FD-895 reduced the expression of Wnt signaling pathway-associated transcripts including *LEF1*, *FN1*, *CCND1*, GSK3β, and *LRP5*. LEF1 is a transcription factor that belong to the T cell Factor (TCF)/LEF family. LEF1 acts as nuclear effector in the Wnt/β-catenin signaling pathway [72]. LEF1 mediates Wnt signaling pathway by through association with β-catenin [46].

Wnt/β-catenin pathway requires the co-receptors LRP5 and LRP6 for activity. Post-translational modifications (PTMS) such as phosphorylation, methylation, acetylation, and sumoylation play a very important role in the pathophysiology of different malignancies. *LRP5* is part of the signalosome complex is deregulated by cisplatin [73]. We also observed the same effect as we saw the non-intronic form of the *LRP5* vanished upon treatment with cisplatin.

Phosphorylation of LRP6 is crucial for activation of Wnt/β-catenin signaling as it can promote activation of Wnt signaling activation by recruiting casein kinase family proteins [74]. Targeting of LRP6 phosphorylation can inhibit Wnt/β-catenin signaling [46].

The co-receptors for Wnt signaling, LRP5, and LRP6, have been revealed as potential oncogenic proteins. In human breast carcinoma, the expression of LRP6 is high [75]. Downregulation of LRP6 inhibits breast cancer tumorigenesis, whereas overexpression of LRP6 in the mouse mammary gland induces mammary hyperplasia [75, 76]. We observed that FD-895 downregulated LRP6 phosphorylation and causes the degradation of the LRP6 protein, an essential component of the Wnt receptor complex, promotes β-catenin degradation, and downregulation of LEF1 at the protein level. Therefore, we reasoned that FD-895 might block the phosphorylation of LRP6 that is required for initial Wnt signaling.

Glycogen synthase kinase-3 (GSK3) is an intracellular component of the Wnt pathway that can directly interact and phosphorylate LRP6. Glycogen synthase kinase 3β (GSK3β) is a crucial component of insulin and Wnt signaling pathways. PTMs particularly phosphorylation of EGFR in lung adenocarcinoma, and SF3B1 in CLL has been reported/targeted [31, 77]. We found that treatment with FD-895, but not by cisplatin lead to induction of IR in GSK3β. An inactivated GSK3β could led to increased SNAIL activity and poor prognosis in cervical cancer [78].

Our results suggest that FD-895 not only modulate RNA splicing *via* induction of IR in genes such as *DNAJB1*, but also inhibit Wnt/β-catenin pathway *via* downregulation of β-catenin, LRP6, pLRP6, and LEF1. FD-895 can block LRP6 phosphorylation and cause degradation of LRP6 protein. Overall this suggests that LRP6 may also serve as a viable anticancer agent in these cell lines, particularly molecules that are capable of selectivity altering the phosphorylation of LRP6. Gene-gene interaction is very crucial for any pathway including Wnt signaling to study the interaction between them. We used GeneMANIA analysis to study the gene-gene interaction prediction with high accuracy [79], identified peripheral nodes corresponding to two genes. The output of analysis led to the identification of gene interactions involved in Wnt signaling (*Wnt3A, CTNNBIP1, DKK1,* and *DKK4*), as well as in RNA splicing (*SF3B6*, and *NELFE*). Among those, there were genes such as *SF3B6*, a gene indirectly interact with *SF3B1* is an integral part of the spliceosome complex. *SF3B6* has been reported to be associated with p53 activity in human non-small cell lung carcinoma [80]. The applications of SPLMs such as E7107, and H3B-8800 have been explored in different clinical trials for therapeutic purposes. The phase I clinical trial at M D Anderson enrolled 26 patients with solid tumors who received escalating doses starting 0.6 mg/m^2^ intravenous infusion on days 1 and 8 and the cycle was repeated every 21 days. The stable disease was observed in 31% of patients. The major AE was the development of acute blindness, which led to the discontinuation of the trial [28]. Similar AE was observed in the Dutch trial on E7107, where reversible grade 4-blurred vision was observed in one patient [29]. Both E7107 trials were suspended due to blindness as one of the AE. H3B-8800, which is an orally available small molecule modulator that binds to SF3B-complex and leads to changes in the alternate RNA splicing in the target cells [81], and induce cell death in the spliceosome-mutant cancers [82] *via* IR of GC-rich introns that are enriched for genes encoding spliceosome components. H3B-8800 approved for testing in Phase 1 (NCT02841540), open-label, first-in-human (FIH) study design to evaluate the safety, tolerability, pharmacokinetics (PK), pharmacodynamics (PD), and preliminary antitumor activity in MDS, AML, and CMML. Additionally, the application of PLAD-B and FD-895 at *in vitro* and *in vivo* levels showed promising results in current and previous studies by different groups. These studies are clinically relevant because one hand where nM concentration is required to achieve IC_50_ in the cancerous cell lines. In contrast, supra-physiological concentration is necessary for normal cells, suggesting a good therapeutic window for treatment options using these SPLMs in conjunction with the current chemotherapy like cisplatin and etoposide. As far as the standalone versus combination therapy is concerned, though we have not assessed the potential of FD-895/Pladienolide- B in combination with an inhibitor of BCL-2 family members, but at the same time there are studies where a combination of splicing inhibitors meayamycin B with ABT-737 (Bcl-xL inhibitor) led to apoptosis in non-small cell lung cancer cell lines (A549 and H1299) [83]. A combination of other SPLMs spliceostatin A (SSA) with either ABT-263 or ABT-199 (both are Bcl-2/Bcl-xL antagonists) led to apoptosis in CLL-B cells [17]. Another example of combination approach where either Sudemycins C, D1, or D6 with BTK inhibitor (ibrutinib) inhibitor led to induction of cell death in CLL-B cells further suggests that synergistic effect can be achieved by combining the SPLMs with different inhibitors to induce cell death *via* modulation of signaling and apoptotic machinery associated pathways. For using stand-alone SPLMs, it is important to know the effect of the SPLMs on the genes/transcripts essential for normal/vital functioning of the body as in the past using E7101 in two clinical trials acute blindness was observed in patients with different malignancies [28, 29].

Collectively, the outcome of the trials, and data generated using other SPLMs; suggest that there is potential in modulating or selectively inhibiting the spliceosome machinery to achieve therapeutic potential in different malignancies including hematologic and solid cancers.

## CONCLUSION

In summary, our results suggest that both FD-895 and pladienolide B demonstrated *in vitro* toxicity in different malignancies, spared normal PBMCs, and modulates mRNA splicing. It also exerts selective toxicity to malignant cells compared with normal cells. Furthermore, we showed that FD-895 (pladienolide B was not explored) able to modulate the post-translational events as suggested by downregulation of LRP6 phosphorylation and expression of associated Wnt target genes. This data suggests that these splice modulators could be useful in targeting malignancies where Wnt/β-catenin play an important role by inhibiting mRNA splicing and LRP6 phosphorylation. These results showed that these compounds not only modulate mRNA splicing in CLL, but also in mantle cell lymphoma, and solid tumors of colon, breast, ovarian, and renal origin. Overall this study demonstrates that FD-895 and pladienolide B modulate splicing machinery and result in downstream regulation of signaling pathways, including the Wnt/β-catenin pathway. Furthermore, the *in vitro* efficacy of FD-895 and pladienolide B was found to be superior to conventional chemotherapy as indicated in a wide range of malignant cell lines of colon, breast, ovarian, renal, and cervical origin. Further, there is a need for extensive research not only at *in vitro* but *in vivo* levels to assess synergistically the ability of splice modulators with conventional chemotherapy agents like cisplatin and etoposide to assess their potential as a combination or synergistic treatments.

## Supplementary Materials

Supplementary Figure 1
