# Supplementary material for: Modulation of RNA splicing associated with Wnt signaling pathway using FD-895 and pladienolide B
Source: Aging (Albany NY). 2022 Mar 1;14(5):2081–100. doi: 10.18632/aging.203924 (PMC8954975; doi:10.18632/aging.203924)
Supplement: Supplementary Figure 1 [file aging-14-203924-s001.pdf]

SUPPLEMENTARY FIGURE

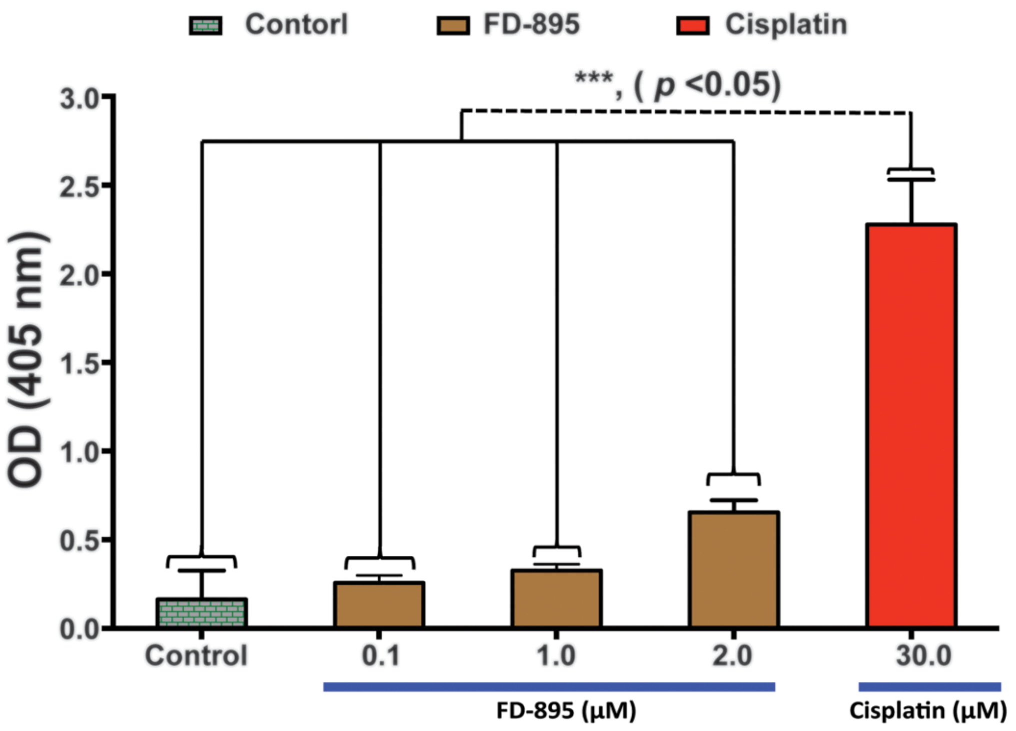

Supplementary Figure 1. *In vitro* cytotoxicity induced by FD-895 and Cisplatin in HEK-293 cell line. HEK cells were exposed to FD-895 (100 nM to 2 μM), and cisplatin (30 μM) for 48 h. Apoptosis was measured in HEK-293 cells using MTS assay.
